# A clinical study of cutaneous leishmaniasis in a new focus in the Kurdistan region, Iraq

**DOI:** 10.1371/journal.pone.0217683

**Published:** 2019-05-31

**Authors:** Nawfal R. Hussein, Amer A. Balatay, Zana S. M. Saleem, Shiraz M. Hassan, Mahde S. Assafi, Ramzy Sh. Sheikhan, Farasheen R. Amedi, Shivan S. Hafzullah, Mahmood S. Hafzullah, Abdulkareem M. Xedr, Mohamed T. Zebary, Hindreen A. Aqrawi

**Affiliations:** 1 College of Medicine, University of Zakho, Zakho, Kurdistan Region, Iraq; 2 College of Pharmacy, University of Duhok, Duhok, Iraq; 3 Department of Internal Medicine, College of Medicine, University of Duhok, Duhok, Iraq; 4 Department of Biology, College of Science, University of Duhok, Duhok, Iraq; 5 Infectious Disease Unit, Azadi Hospital, Directorate of Health, Duhok, Iraq; 6 Department of Pathology, Duhok Central Laboratory, Duhok, Iraq; Academic Medical Centre, NETHERLANDS

## Abstract

Leishmaniasis is caused by protozoan parasites of the genus *Leishmania* and is a major health problem in various parts of the world. Cutaneous leishmaniasis (CL) occurs, among others, in unpredictable outbreaks after wars and disasters. After the last war in Iraq, the collapse of the health system led to the spread of infectious diseases, including CL. Between September 2016 and November 2017, all patients with confirmed CL having one or more skin lesion(s) were referred to a regional health center in Musol City within the Rabeea District. During this period, 1539 patients visited the clinic. A total of 190 patients were excluded from the study because of poor follow-up. The treatment success rate was 97.7% (1319/1349). Statistically significant associations were found between number of lesions and treatment failure (p = 0.0018; OR = 1.4430; CI = 1.1466–1.8161), number of doses and number of lesions (Pearson’s correlation coefficient = 0.095; p = 0.001), and the lack of municipality services and number of doses used for the treatment (p = 0.008; OR = 1.0629; CI = 1.0158–1.1122). To conclude, the highest number of patients with CL in the city of Musol was recorded after the war in Iraq. The treatment success rate was high, which reflected the strict treatment and follow-up program. An urgent plan is needed to stop the spread of infection.

## Introduction

Leishmaniasis is an old protozoan parasitic disease that is caused by *Leishmania* species [1, 2]. The infection is widespread in approximately 100 countries, and more than 10 million people are infected with the parasite [1, 2]. Additionally, 350 million people are at risk of acquiring the infection [1, 2]. Leishmaniasis can present itself in three forms: cutaneous leishmaniasis (CL), mucocutaneous leishmaniasis (MCL), and visceral leishmaniasis (VL)[3]. it can cause significant mortality and morbidity [4], and is considered a neglected disease [5]. Nevertheless, the pharmaceutical industry seems reluctant to invest in research and development of new medications due to the lack of sufficient incentives [1, 2]. CL is one of the most common zoonotic infectious diseases caused by 15 different species of the protozoan parasite. More than two-thirds of CL cases occur in the Middle East, Central Asia and South America [3]. CL may lead to a scar that causes mental, social and emotional issues, in particular for women and children [1, 2]. To combat the infection, a better understanding of the epidemiology of the disease is needed in each country.

Therefore, the World Health Organization (WHO) has recommended activities in the field of research regarding the epidemiology, diagnosis, and treatment of CL in endemic countries [4]. Both CL and VL are endemic in Iraq. The maximum number of CL cases was reported after the Second Gulf War in 1992, reaching 45.5 cases per 100,000 people [5]. Then, the trends of infection started to decline until 2014 when ISIS occupied wide territories of Iraq, leading to failure of the health system. Additionally, with internal displacement of people, there was a fair chance that infectious diseases spread to new areas. This study was conducted to investigate the epidemiological status of CL in Musol city, Rabeea district, and evaluate treatment outcome and factors influencing the outcome.

## Patients and methods

### Patients

All patients who were diagnosed with CL and had given consent were included in this study. Patients who had received treatment from more than one clinic and who did not follow the protocol of this study were excluded. In this study, patients with CL, who were referred to the health center in the Rabeea District, city of Musol, between September 2016 and November 2017, were recruited. Data regarding age, gender, occupation, place of residence, and the number of lesions were recorded.

### Treatment protocol and outcome

Patients with 3 or fewer lesions and with a diameter of 3 cm or more were treated with intralesional injections twice weekly for a total of 10 injections. For patients with more than 3 lesions and with diameters greater than 4 cm, or with lesions at sites difficult to assess (such as ears, eyelids, nose), intramuscular injections were given on a daily basis for two weeks with a break of two days for a weekend. Patients were treated with Pentostam at a dose of 20 mg/kg/day (sodium stibogluconate, England) [6]. Patients were followed-up weekly for 6 months after the treatment. During the follow-up sessions, the lesions were carefully examined. Based on clinical examination of the lesion, subjects were classified into two possible outcomes: first, cured, which was defined as complete wound healing with reepithelization and the absence of any sign of activity or inflammation. Second, treatment failure was defined as the presence of signs of inflammation around the wound or the appearance of new lesions. When the lesion size decreased with less inflammation, the status was considered pending.

### Statistical analysis

Linear regression was used to explore the relationship between variables and outcomes when the variables were continuous, while the chi square test was used for categorical data. To explore the effect of variable factors on the number of doses given to each patient per lesion, a Persons correlation study was conducted. Statistical analysis was carried out with Minitab 18. P values < 0.05 were taken to indicate statistically significant differences.

### Ethics

The study protocol and informed consent were approved by the Research Ethics Committees of the College of Pharmacy, University of Duhok and the Directorate of Health in Duhok City, Kurdistan Region, Iraq. Informed written consent was obtained from all recruited subjects or their legal guardians.

## Results

### Epidemiologic status

During the study, 1539 patients with CL visited our clinic. A total of 190 patients were excluded from the study because they did not visit the clinic regularly or received treatment from more than one clinic. The majority of the patients was adults older than 18 years with a male to female ratio of 1:1. While 47.3% of the patients presented within the first month of the appearance of the lesions, 6.7% of the subjects presented after 6 months of the appearance of the lesions (Table 1).

**Table 1:** Patient characteristics.

Age group	Number	Percentage
0–6	145	10%
6 to 12	373	24.2
12 to 18	234	15.2
18 to 40	542	35.2
Older than 40	236	15.3
Duration of disease in days		
0 to 30	730	47.4
30 to 90	704	45.8
90 to 120	2	0.2
More than 120	103	6.7
Number of lesions		
1	1033	67.1
2	297	19.3
3	127	8.3
4	52	3.4
More than 4	30	1.9
Job		
Housewife	363	23.6
Student	285	18.5
Worker	151	9.9
Farmer	41	2.7
Soldier	54	3.5
Police	9	0.6
Other	636	41.2
Gender		
Male	784	50.9
Female	755	49.1

### Treatment outcome

In this study, 1349 patients were evaluated for response or treatment failure. Among these, 540 received intramuscular injections. A total of 533/540 (98.7%) patients who received intramuscular injections showed a complete response to treatment. On the other hand, 809 patients received intralesional injections. Among those who received intralesional injections, 786/809 (97.2%) were cured completely. The overall treatment success rate was (1319/1349) 97.7%.

### Factors affecting cure

We studied the effect of different variables on the outcome of treatment. Linear regression was used to explore the relationship when the variables were continuous, while the chi square test was used for categorical data. A statistically significant association was found between the number of lesions and treatment failure (p = 0.0018; OR = 1.4430; CI = 1.1466–1.8161). No statistically significant association was found between age, duration of the lesion, gender, the presence of livestock, or the availability of the municipal services on the one hand, and treatment failure on the other hand (Table 2).

**Table 2:** Factor affecting treatment outcome.

	Cured	Treatment failure	P value	OR	CI 95%
Age	15.4±16.1	18.8±11.5	0.1	1.01	0.99–1.03
No. of lesions	1.3±0.54	2.2±1.4	0.001	1.44	1.14–1.81
Duration of lesion	75±40.8	58±35	0.23	1	0.99–1.01
Gender (female)	644/1319 (48.8)	12/30 (40%)	0.35	1.42	0.68–2.99
Livestock	957/1319 (72.6%)	25/30 (83.3%)	0.19	1.9	0.72–5
Municipality services	784/1319 (59.4%)	15/30 (50%)	0.3	1.46	0.70–3.01

### Factor affecting the number of doses

To explore the effect of variable factors on the number of doses given to each patient per lesion, the Pearson’s correlation coefficient was used. No correlation was found between the number of doses and the duration of the lesion (Pearson’s correlation = 0.005; p = 0.8). A statistically significant association was found between the number of doses and the number of lesions (Pearson’s correlation = 0.095; p = 0.001). No association was found between the number of doses and the age of patients (Pearson’s correlation = 0.015; p = 0.59). Linear regression was used to assess the effect of gender, the presence of livestock, or the availability of the municipal services on the one hand, and the number of doses on the other hand.

A statistically significant association was found between the lack of municipal services and the number of doses used for treatment (p = 0.008; OR = 1.0629; CI = 1.0158–1.1122).

## Discussion

During our study period, 1482 cases of CL were recorded over 12 months in Musol City, Rabeea District. To our knowledge, this is the highest ever reported number of cases in a single province in Iraq. This could be attributed to a significant decline in health services suffered postwar in addition to poor sanitary conditions, which may have led to a rise in vector populations. In addition, internal displacement of people from endemic areas might have played a role in the increase in leishmaniasis cases. In Iraq, the prevalence of CL peaked after the second Gulf War in 1991, when the health system momentarily collapsed. Approximately 10,000 CL cases had been reported between 1991 and 1997 [5]. Then, the number of cases declined during the following years until 2014, when there was widespread occupation of the country by ISIS. In 2013, approximately 2000 cases were reported in all of Iraq. The highest number of reported cases in a single province was 400 in Rahmania Province [5].

In this study, two forms of treatment were used: intralesional and intramuscular injections. The overall treatment success rate was 97%, which was higher than that reported previously in Iraq (80%) [5]. The high success rate could be attributed to positive factors, such as good public education programs, strict patient follow-up, and more aggressive patient outreach in difficult to reach areas. Various factors have been shown to influence the pathogenesis and treatment outcome of CL. A study involving animals demonstrated that host, gender, and genotype influenced the immune response to the parasite and induced more severe signs and symptoms in females [7]. However, in clinical studies, gender did not influence the treatment outcome [4, 8, 9]. In agreement with this, in our study, gender did not impact the outcome of treatment. Additionally, it was previously found that treatment failure was associated with the number of lesions [10–12]. In our study, we found a significant correlation between a higher number of lesions and treatment failure. This might be explained by the higher parasite load found in patients carrying a higher number of lesions and/or by the coexistence of different genotypes of the parasite. Further studies are needed to investigate the genotypes of the parasite in Iraqi patients.

Previous studies have also shown that treatment failure occurred more often in children [13, 14]. This was explained by the immaturity of the immune system in such an age group [13, 14]. However, such a correlation was not observed in our study.

Some evidence suggests that early treatment of CL, before the development of effective acquired immunity, is associated with treatment failure as the initial response to the parasite is nonspecific and not enough to eradicate the microorganism [12]. In support of this, previous studies suggested that treatment in the early stage was associated with an increased failure rate. We did not find a significant association between the duration of infection and cure rate. Additional studies are needed to investigate the immune response and disease development process and to determine the best time for treatment initiation.

Municipal services, including garbage containers, were not available in all districts from which our patients came. The lack of such services might lead to the accumulation of household waste, which in turn could create an excellent environment for the sandfly to grow and multiply. We found a significant association between the absence of municipal services and higher doses of treatment. This might be explained in part by reinfection during the treatment.

Our study has limitations. First, we could not register all cases in Musol City due to geographical barriers and the closure of main roads due to the war. Second, a lack of adequate funding prevented us from having further follow-up and more aggressive management of cases that failed to respond to initial treatment.

## Conclusions

To conclude, the collapse of health services after the last war in Iraq led to a spike in the cases of LC. We reported the greatest number of patients with CL in Musol. In this study, the cure rate was higher than previously reported in the same region. This may be due to the strict follow-up programs and more aggressive management. Factors affecting treatment were investigated, and a statistically significant association was found between the number of lesions and treatment failure. A significant association was found between the number of lesions, lack of municipal services and the number of doses required for the treatment of LC. To control the infection, active and passive surveillance must be established alongside making the treatment more readily available and free of charge. Multisectoral activities are needed to control the vector and reservoir, promote health education, conduct research and increase community participation in combating LC infection.
